# Ferroptosis: Potential therapeutic targets and prognostic predictions for acute myeloid leukemia (Review)

**DOI:** 10.3892/ol.2024.14707

**Published:** 2024-09-30

**Authors:** Wenlu Zhang, Wen Wen, Ran Tan, Meirui Zhang, Tantan Zhong, Jianhong Wang, Haiping Chen, Xiaosheng Fang

**Affiliations:** 1 Department of Hematology, Shandong Provincial Hospital Affiliated to Shandong First Medical University, Shandong First Medical University, Jinan, Shandong 250021, P.R. China; 2 Department of Hematology, Shandong Provincial Hospital Affiliated to Shandong First Medical University, Jinan, Shandong 250021, P.R. China; 3 Department of Infectious Diseases, Shandong Provincial Hospital Affiliated to Shandong First Medical University, Jinan, Shandong 250021, P.R. China

**Keywords:** ferroptosis, acute myeloid leukemia, lipid metabolism, iron metabolism, ferroptosis mechanism

## Abstract

Ferroptosis is a relatively recently discovered type of regulated cell death that is induced by iron-dependent lipid peroxidation. The key contributing factors to ferroptosis are the loss of glutathione peroxidase 4 which is required for reversing lipid peroxidation, the buildup of redox-active iron and the oxidation of phospholipids containing polyunsaturated fatty acids. Ferroptosis has been associated with a number of diseases, including cancers such as hepatocellular carcinoma, breast cancer, acute renal damage and neurological disorders such as Alzheimer's disease and Alzheimer's disease, and there may be an association between ferroptosis and acute myeloid leukemia (AML). The present review aims to describe the primary regulatory pathways of ferroptosis, and the relationship between ferroptosis and the occurrence and development of AML. Furthermore, the present review comprehensively summarizes the latest advances in the treatment and prognosis of ferroptosis in AML.

## 1. Introduction

AML is a malignant clonal proliferative disease of hematopoietic stem cells. The diagnosis and therapy of AML have advanced in recent years due to continuous research and an improved understanding of the etiology of the disease (1). Numerous targeted small-molecule inhibitors have been approved for the treatment of AML, which have demonstrated favorable curative results, including chemotherapy and hematopoietic stem cell transplantation (2). AML has a poor prognosis and a high rate of recurrence (1,2). Thus, further research to explore novel therapeutic drugs to improve the prognosis of AML is required. The diagnosis of AML is primarily dependent on clinical presentation and pathological features, particularly the presence of specific gene mutations, such as RUNX1 and TP53, which are often associated with poor prognosis. In addition, according to World Health Organization (WHO) standards, the diagnosis of AML requires that the proportion of primitive cells (blastocytes) in the bone marrow or peripheral blood be at or above 20%. Comprehensive analysis of these factors is essential for accurate diagnosis and effective treatment planning (3).

With an increasing understanding of iron metabolism and the identification of ferroptosis as a novel type of regulated cell death dependent on iron, novel therapeutic options have emerged in recent years (4,5). The ability to induce effective tumor cell death, while protecting healthy cells is critical for cancer therapy. For tumor survival and growth, cancerous cells require larger quantities of iron than healthy cells (6). Given their dependency on iron, tumor cells are more vulnerable to iron-induced apoptosis (7,8). Regulation of the ferroptotic pathway to prevent the development and growth of malignancies has garnered increasing interest in recent years (9–11). The present review aims to provide an overview of ferroptosis in the incidence, development, prognosis and as a potential therapeutic target of AML.

## 2. Mechanisms of ferroptosis

Ferroptosis, a unique type of cell death characterized by the buildup of lipid reactive oxygen species (ROS) and iron dependency, was initially described by Dixon *et al* (12) in 2012. Ferroptosis describes a mode of cell death distinct from apoptosis, necrosis and autophagy. Ferroptosis is characterized by reduced mitochondrial volume, increased lipid bilayer density, and diminished or absent mitochondrial cristae, while the cell membrane and the nuclear morphology remain unaffected (13,14). In biological terms, lipid peroxide metabolism is catalyzed by reduced glutathione peroxidase 4 (GPX4) activity and intracellular glutathione (GSH) levels (15). Ferroptosis is induced by the Fenton reaction, in which Fe^2+^ oxidizes lipids and generates significant quantities of ROS (16,17).

**Figure 1.**
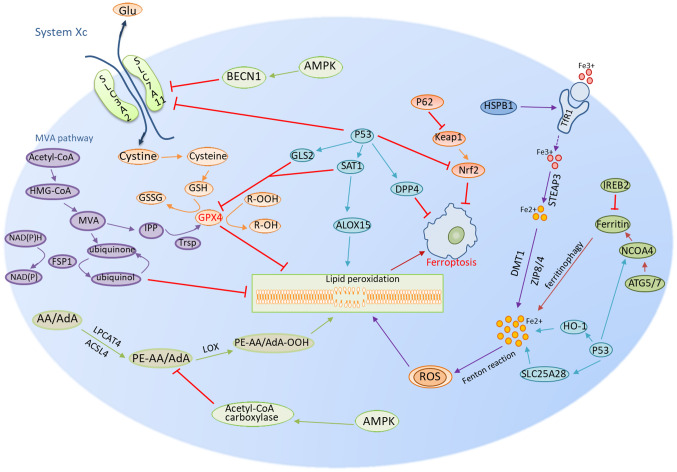
Mechanisms of ferroptosis encompass a number of pathways, including those related to GSH/GPX4, iron metabolism and lipid metabolism. The GSH/GPX4 pathway includes system Xc and the MVA pathways and part of the p53 pathway. Iron metabolism is the second group, which involves other regulatory mechanisms in part of the Nrf2, NCOA4, IREB2, HSPB1 and the p53 partial pathway. The lipid metabolism route is the third group, involving factors such as p53-SAT1-ALOX15, ACSL4 and LPCAT3. AMPK signaling represents the fourth class of regulatory mechanisms involved in ferroptosis (A single arrow means promotion of the process and a red T arrow means inhibition of the pathway. Different colors represent different metabolic processes). GSH, glutathione; GPX4, glutathione peroxidase 4; GSSG, oxidized glutathione; SLC7A11, solute carrier family 7 member 11; SLC3A2, solute carrier family 3 member 2; R-OOH, lipid hydrogen peroxide; R-OH, lipid alcohols; MVA pathway, mevalonate pathway; HMG-CoA, hydroxymethylglutaryl-CoA; IPP, isopentenyl pyrophosphate; TRSP, selenocysteine-specific transfer RNA; FSP1, ferroptosis suppressor protein 1; GLS2, glutaminase 2; SAT1, spermine N1-acetyltransferase 1; LOX, lipoxygenase; ALOX15, arachidonate-15-lipoxygenase; BECN1, beclin 1; DPP4, dipeptidyl peptidase-4; Keap1, ECH associated protein 1; Nrf2, nuclear factor erythroid 2-related factor 2; HSPB1, heat shock protein family B (small) member 1; TfR1, transferrin receptor 1; STEAP3, six-transmembrane epithelial antigen of prostate 3; STEAP family member 3; DMT1, divalent metal transporter 1; ZIP8/4, zinc transporter ZIP8/4; NCOA4, nuclear receptor coactivator 4; ATG5/7, autophagy protein 5; HO-1, heme oxygenase 1; SLC25A28, solute carrier family 25 member 28; AMPK, AMP-activated protein kinase; AA, arachidonic acid; AdA, AdA, adrenoyl derivatives; PE-AA/AdA, phosphatidylethanolamines; Acetyl-CoA, acetyl coenzyme A; LPCAT3, lysophospholipid acyltransferase.

Ferroptosis mechanisms can be broadly classified into four categories: i) The GSH/GPX4 pathway, ii) the inhibitory system Xc, iii) the mevalonate (MVA) pathway; and iv) p53 regulation (Fig. 1) (13). The solute carrier family 7 member 11 (SLC7A11) and SLC3A2 subunits constitute the amino acid anti-transporter system Xc, which is present in the cell membranes of various cell types (18,19). Glutamate and cystine are imported and exported by system Xc in a 1:1 ratio. The cystine that is imported into the cell is reduced to form GSH. In the presence of GPX, GSH can reduce ROS. GPX4 is a crucial regulator of iron-induced mortality in the GPX family (20). GPX4 converts lipid hydrogen peroxide (R-OOH) to lipid alcohols (R-OH), which limits iron-dependent lipid ROS generation and protects membrane integrity from damage induced by ROS buildup (21). The MVA pathway synthesizes isoprene units such as isopentenyl pyrophosphate (IPP) and dimethylallyl pyrophosphate (DMAPP) from acetyl coenzyme A (acetyl-CoA) (22). This pathway also contributes to stabilization of the transport RNA (tRNA) needed for selenocysteine incorporation into selenoproteins, through protein isoprenylation. A key selenoprotein affected is GPX4, which clears toxic lipid peroxides to suppress oxidative cell damage. Inhibition of the MVA pathway, by statins for example, reduces IPP/DMAPP production and selenoprotein synthesis, which reduces GPX4 activity (23,24). This impairs the elimination of lipid peroxides and sensitizes cells to ferroptosis. However, an impaired mevalonate pathway also reduces the synthesis of coenzyme Q10, an antioxidant that partners with ferroptosis suppressor protein 1 (FSP1) and NADH to neutralize the toxic lipid radicals that accumulate in cells. Thus, the coenzyme Q10-FSP1-NADH system acts in an anti-ferroptotic manner, downstream of mevalonate pathway inhibition to delay the induction of iron-mediated oxidative cell death (25).

Ferroptosis is regulated by p53 via two mechanisms (26,27). Ferroptosis has been reported to be inhibited by p53 in colorectal cancer cells through the formation of a dipeptidyl peptidase-4 (DPP4)-p53 complex and the subsequent translocation of the DPP4 enzyme from the cell membrane to the nucleus. This process reduces the activity of DPP4 on the cell membrane, thereby decreasing lipid peroxidation and ultimately inhibiting ferroptosis (28). p53-mediated inhibition of ferroptosis also increases SLC7A11 expression levels through the inhibition of Nrf2-mediated gene expression, which leads to increased GSH synthesis and lipid peroxide clearance (29,30). p53 can also contribute to ferroptosis through several approaches: i) The suppression of system Xc, which reduces cystine uptake and GSH production (28); and ii) transcriptional activation of glutaminase-2 to decrease GSH levels and GPX4 activity (27). The latter mechanism is related to iron metabolism, such as via the p62-kelch like ECH associated protein 1 (Keap1)-nuclear factor erythroid 2-related factor 2 (Nrf2) regulatory pathway, nuclear receptor coactivator 4 (NCOA4) pathway that is related to ferritin metabolism, iron responsive element binding protein 2 (IREB2), p53 and heat shock protein family B (small) member 1 (HSPB) regulatory pathways (13). Intracellular iron levels are crucial in order to maintain intracellular homeostasis and increased iron levels can induce a Fenton reaction, which results in the formation of intracellular ROS. Lipid peroxidation processes can be induced by excess levels of ROS and lead to ferroptosis (31). The transferrin receptor 1 (TfR1) is a membrane protein that binds to the Fe^3+^/transferrin complex, which allows iron to be released into endocytic vesicles inside the cell (9). Once inside the vesicles, Fe^3+^ is reduced to Fe^2+^ by the iron reductase enzyme, STEAP. Fe^2+^ then enters the cytosol, creating an unstable pool of free iron known as the labile iron pool (LIP) (32). The LIP is regulated by either the zinc-iron regulatory proteins ZIP8 and ZIP14, or the divalent metal transport protein 1 (33). The iron from the LIP can be stored inside the protein ferritin or transferred back out of the cell via the iron export protein ferroportin (FPN) to keep the LIP levels low and prevent iron-induced cell death. Through the utilization of TfR1 and FPN, cells can control iron import and export to meet its needs while avoiding excess free iron that could cause oxidative damage (34). In 2016, Sun *et al* (35) demonstrated that the p62-Keap1-Nrf2 pathway controls the suppression of ferroptosis in hepatocellular carcinoma. The antioxidant response is notably regulated by Nrf2, which can reduce ferroptosis and improve tumor chemotherapy response and radiation resistance (36). Keap1 targets Nrf2 for ubiquitination and degradation, thus inhibiting Nrf2 (37,38). p62 is a multifunctional autophagy receptor, and the accumulation of p62 reduces the inhibition of Keap1 and promote the release of Nrf2 (39).

Within the NCOA4 pathway, a process known as ferritin phagocytosis, which is mediated by the cargo receptor NCOA4, can cause a subsequent release of iron into the cytoplasm to reduce iron toxicity (40). Ferritin is made up of the ferritin heavy chain 1 (FTH1) and the ferritin light chain (FTL) (41). Erastin-induced ferroptosis was inhibited by increasing the expression levels of FTL and FTH1 while significantly decreasing the expression levels of IREB2, a crucial transcription factor of iron metabolism (42,43). Heat shock protein family B (small) member 1 (HSPB1) overexpression can further raise intracellular iron content by increasing TRF1 expression and preventing ferroptosis (44). Previous studies have uncovered additional mechanisms by which p53 regulates ferroptosis by modulating iron metabolism and availability (45). p53 promotes ferroptosis by transcriptionally activating TfR1 and the mitochondrial iron importer solute carrier family 25 member 28 (SLC25A28), which results in increased uptake and accumulation of reactive iron, that sensitizes cells to oxidative damage (46). In addition, p53 can drive the degradation of the iron-storage protein ferritin by activating NCOA4. This results in the release of stored iron to further increase reactive intracellular iron levels (47). Conversely, p53 can also increase the expression of heme oxygenase-1 (HO-1) which reduces overall cellular iron levels. By decreasing iron availability, HO-1 activity inhibits lipid peroxidation and suppresses ferroptosis sensitivity (48).

The third mechanism is related to lipid metabolism pathways, such as the p53-SAT1-arachidonic acid lipoxygenase 15 (ALOX15), ACSL4 and lysophospholipid acyltransferase (LPCAT3) pathways (9). The process of lipid peroxidation is crucial to ferroptosis. Spermine N1-acetyltransferase 1 (SAT1) is the transcriptional target of p53 in the p53-SAT1-ALOX15 pathway, and activation of SAT1 can induce lipid peroxidation to encourage ferroptosis, which is tightly connected through ALOX15 control (13). Lipid peroxidation is initiated by the formation of arachidonic acid (AA)/adrenoyl derivatives (AdA). Polyunsaturated fatty acids (PUFAs) such as AA and AdA are esterified to form PUFA-PL complexes (PE-AA/AdA) under the action of ACSL4 and LPCAT, resulting in the accumulation of PE-AA/AdA in the cell membrane to produce lipid peroxides via LOX activity and Fenton reactions, which induce ferroptosis (49). The enzymatic pathway utilizes LOXs, which are non-heme iron-containing dioxygenases. PE-AA/AdA serve as substrates for 15-LOX to form phospholipid hydroperoxides (PE-AA/AdA-OOH) (36,40). These peroxyl radicals propagate further peroxidation of neighboring PUFAs (32). In addition, in the non-enzymatic pathway, free redox-active iron reacts with PE-AA/AdA via Fenton reactions to generate membrane-destabilizing lipid peroxides (50). In both pathways, the iron-dependent peroxidation of membrane PUFAs catalyzed by PE-AA/AdA intermediates results in excessive free radical damage, depletion of antioxidants, loss of membrane integrity and ultimately, execution of regulated ferroptotic cell death (9,51).

Finally, there are other regulatory pathways involved in ferroptosis, such as the AMP-activated protein kinase (AMPK) signaling pathway. AMPK acts as a sensor of the cellular energy status and contributes to the preservation of energy homeostasis (52). To prevent PUFA biosynthesis and ferroptosis, AMPK can mediate the phosphorylation of acetyl-CoA carboxylase. However, AMPK also inhibits SLC7A11-mediated cystine transport and increases ferroptosis by modulating the phosphorylation of beclin1 (BECN1) (15,53).

## 3. Role of ferroptosis in the development and progression of AML

AML is a heterogeneous disease characterized by constitutively activated oncogenic signals, high mortality rates and a poor prognosis. It is typically treated with chemotherapy and patient survival rates are poor (54). Therefore, novel therapeutic approaches are required to improve the management of the disease (55). Iron serves a role in hematopoiesis and is a necessary component of human cells. After interacting with the transferrin-iron complex, the membrane protein TfR1 releases iron via endocytosis. When TfR1 expression is suppressed, iron deprivation reduces the ability of hematopoietic stem cells to regenerate and affects the proliferation and differentiation of hematopoietic progenitor cells. ROS accumulation, induced by excessive iron, can result in oxidative stress which can damage proteins, DNA, lipids and even induce cell death (14,53). Patients with AML frequently exhibit severe symptoms resulting from iron overload, primarily due to the extensive blood transfusions required to manage anemia caused by abnormal erythropoiesis and chemotherapy. These symptoms include heart failure, liver fibrosis, diabetes, endocrine disorders, fatigue, and weakness, significantly impacting the patients' quality of life and prognosis (40). Additionally, the rapid proliferation of specific hematopoietic precursor cells in patients with AML leads to an increased demand for iron (56). Thus, given the tendency for iron-overload and the iron dependency of AML, the induction of ferroptosis represents a promising therapeutic approach (40,56).

GPX4 is an antioxidant enzyme that uses GSH to transform toxic lipid peroxides into non-toxic lipols, thereby protecting cells from ferroptosis. Previous studies (49,57) have demonstrated that GPX4 is upregulated in AML cells, which enables cells to restrict ferroptosis through reduced lipid peroxide levels, which ultimately increases AML cell survival and drug resistance (58). Consequently, GPX4 serves a critical role in AML progression and represents a promising therapeutic target. The survival pathway of cancer cells, particularly the antioxidant response regulated by Nrf2, serves a crucial role in the defense against apoptosis, which is a key factor in cancer cell survival (59). Nrf2 has been linked to the development of AML and previous studies suggest that it can be regulated by NF-κB in AML. This activation of the Nrf2-dependent antioxidant defense response provides growth advantages and resistance to treatment for AML cells (60).

Lipid metabolism and ferroptosis are intricately intertwined, as the emergence of lipid peroxides is one of the hallmarks of ferroptosis (61). In patients with AML, lipid homeostasis is disrupted, and PUFAs and other unsaturated fatty acids present in AML cells contribute to the onset and progression of the disease (62). Although there is limited research on the mechanisms underlying the development of AML, it is clear that further investigation is necessary to elucidate the specific molecular mechanisms involved (Table I).

## 4. Ferroptosis in AML therapy

AML is the most common type of leukemia in adults and is typically treated with chemotherapy. However, chemotherapy can have severe side effects and cause drug resistance in leukemia cells (63). Although hematopoietic stem cell transplantation can cure AML in a number of cases, its widespread use is limited. For instance, the relapse rate post-transplant is relatively high, particularly among high-risk patients. In addition, patients face significant infection risks during and after the transplantation process. Therefore, there is an urgent need for more effective and accessible treatment for AML. Recent studies have shown that inducers of ferroptosis, a process that involves the buildup of reactive oxygen species (ROS) and lipid peroxidation, have potential to treat certain types of cancer (64,65).

Numerous substances, including eprenetapopt (APR-246), aldehyde dehydrogenase 3 family member A2 (ALDH3A2), poly(lactic acid)-glycolic acid-encapsulated glycyrrhetinic acids (GCMNPs), glutathione-bioimprinted nanoparticles targeting of N6-methyladenosine FTO demethylase (GNPIPP12MA), gold nanorods (GnR) functionalized with chitosan and a 12-mer peptide 12 (GnRA-CSP12) and GCFN, have been found to induce ferroptosis in AML cells by disrupting the balance between GSH and ROS and inhibiting GPX4 synthesis (51,66–69). In a study by Birsen *et al* (66), APR-246 was reported to induce ferroptosis in AML cells by decreasing GSH concentration and increasing ROS and lipid peroxides. This treatment may be effective for different subtypes of patients with AML, as it works regardless of the presence of p53 mutations. It was also reported that ferroptosis inducers, RAS-selective lethal (RSL3) and FINO_2_, a 1,2-dioxolane, increased the antileukemic activity of APR-246 in AML *in vitro* (70,71). In phase II studies of myelodysplastic syndromes/AML with APR-246, adverse neurological events were reported in over one-third of patients who received APR-246 (72). Additionally, previous research has demonstrated an association between ferroptosis and neurological disorders, particularly neurodegenerative disorders (73). ALDH3A2 is an aldehyde dehydrogenase that is present in both healthy myeloid cells and primary AML cells (67). It is essential for the detoxification of aliphatic aldehydes and the synthesis of 16- and 18-carbon fatty acids. Yusuf *et al* (67) reported that AML cells that were deficient in ALDH3A2 had altered biosynthetic pathways, exhibited increased oxidative damage and had an altered cellular lipid composition. It was reported that lipid peroxidation was associated with the induction of ferric death and an increase in lysophospholipids was observed experimentally, particularly in the absence of polyunsaturated fatty acid tails as a marker of ferroptosis. Moreover, ALDH3A2 depletion caused ferroptosis in leukemia cells and acted synergistically with GPX4 inhibition. Furthermore, ALDH3A2 depletion caused iron depletion in leukemia cells and preserved normal hematopoietic function. Hence, the inhibition of ALDH3A2 in combination with ferroptosis inducer drugs, particularly GPX4 inhibitors, may be a potential treatment for AML (67).

CircKDM4C is a cyclic RNA produced by the KDM4C gene. According to Dong *et al* (74), circKDM4C increases p53 expression levels by regulating the microRNA (miRNA) hsa-let-7b-5p. Specifically, circKDM4C acts as a sponge for hsa-let-7b-5p, reducing its inhibitory effect on p53, thereby enhancing p53 expression levels. The increase in p53 expression levels lead to an increase in intracellular iron concentration and ROS expression levels and caused ferroptosis in AML cells. Initially identified as a regulator of cell proliferation in *Caenorhabditis elegans*, miRNA hsa-let-7 was found to be upregulated in AML. Previous research has demonstrated that p53 can block the cystine system by downregulating SLC7A11, which in turn decreases GPX4 activity. This decrease in GPX4 activity led to a decrease in the antioxidant capacity of leukemia cells and an accumulation of ROS, causing ferroptosis (75). As a result, circKDM4C presents a potential target for the treatment of patients with AML.

Yu *et al* (76) developed a ferroptosis-inducing nanotherapeutic drug (GCFN) based on glutathione reactive cysteine polymer. To evaluate the efficacy of GCFN in treating AML, a mouse model of aggressive AML was developed. GCFN could effectively induce lipid peroxidation and ferroptosis in AML cells by reducing the expression levels of intracellular GSH and inhibiting the activity of GPX4, thus providing a basis for GCFN as a potential treatment for AML.

Immunotherapy is a valuable therapeutic method that has been successfully applied in clinical settings. Breaking autoantigen immune tolerance is essential for antitumor immunotherapy (51). Targeted therapies are a promising research strategy because they have been shown to enhance tumor immunity by activating T cells, modulating the tumor microenvironment, enhancing antigen presentation and inhibiting immune checkpoints, thereby improving treatment effectiveness and reducing the likelihood of recurrence (77,78). For example, studies have found that the combination of GCMNPs with PD-L1 blockers may potentially improve efficacy in the management of leukemia (68). GNPIPP12MA enhances anti-leukemia immunity by increasing cytotoxic T cell infiltration (79). GnRA-CSP12 disrupts intracellular REDOX balance, regulates epigenetic transcriptomics, and further enhances cytotoxic response of T cells. These combination therapies have shown promising results in preclinical models, showing great potential to improve the treatment of leukemia (80).

GCMNPs are highly specific to cancer cells and have low toxicity in AML (68). Through the inhibition of GPX4, GCMNPs can induce ferroptosis in AML cells, which increases lipid peroxide levels (51). In this study, a mouse model of AML was successfully used to evaluate the immune response to cancer immunotherapy, particularly against AML and colorectal cancer. Encouragingly, the animals did not experience any weight loss or damage to kidney, heart, liver or lung tissue during treatment. This result not only demonstrates the efficacy of the treatment, but also highlights its safety, providing valuable insights for future cancer immunotherapies. This demonstrates the significant value of the AML mouse model in assessing the efficacy and safety of cancer immunotherapy. Ferumoxytol and GCMNPs can also work together to increase the Fenton reaction and cause ferroptosis. Additionally, the combination of GCMNPs, Ferumoxytol and anti-PD-L1 improved T cell immune responses against leukemia (68).

In AML, fat mass and obesity-associated protein (FTO), an N6-methyladenosine (m6A) demethylase, contributes to carcinogenesis by preventing the expression of immune checkpoint genes, particularly LILRB4. Knockdown of FTO reduced the growth of leukemia stem cells and prevented leukemia cells from escaping the immune system (79). GNPIPP12MA is an FTO inhibitor-loaded GSH-bioimprinted nanocomposite (69). Through the FTO/m6A pathway, GNPIPP12MA induces GSH depletion to inactivate GPX4, inhibit the decrease in LPO, increase intracellular iron accumulation and lead to the selective ferroptosis of AML cells. GNPIPP12MA therefore has a wide range of anti-AML effects at relatively low doses. Additionally, GNPIPP12MA could improve antileukemic immunity by increased infiltration of cytotoxic T cells (69,79).

Nanoparticles of gold hexadecyltrimethylammonium bromide and sodium oleate are used as a binary surfactant combination to create GnRA-CSP12 (80), which are GnRs with various aspect ratios. GnRA-CSP12 is selectively taken up by leukemia cells through targeted endocytosis, disrupting the intracellular redox balance, inducing ferroptosis and regulating epitranscriptomics by eliminating Fe^2+^-dependent m6A demethylase activity. This enhances the cytotoxic response of T cells, thereby improving immunotherapy efficacy. Specifically, GnRs reduced GSH expression levels through the formation of Au-S bonds with GSH, disrupting the GSH/ROS balance and ferroptosis of leukemia cells. In the AML mouse model treated with GnRs, no changes in body weight or pathological alterations in major organs were observed and no significant toxic effects or side effects were detected.

The regulation of ferroptosis also involves a number of signaling molecules and pathways. For example, the quinazolinone derivatives, Erastin and high mobility group box 1 (HMGB1) (81), regulate ferroptosis through the JNK/p38 pathway, while dihydroartemisinin (DHA) and typhaneoside (TYP) regulate ferroptosis through the AMPK signaling pathway (82,83). 4-amino-2-trifluoromethyl-phenyl retinate (ATPR) modulates ferroptosis via Nrf2 signaling (84). Imetelstat influences ferroptosis by modulation of the ACSL4 and FADS2 molecular signaling pathways (85). Finally, Honokiol regulates ferroptosis by upregulating the expression levels of heme oxygenase 1 (HMOX1) (86).

The discovery of Erastin was initially prompted by its ability to selectively induce cell death in cancer cells with mutant RAS (87). In a study conducted by Yu *et al* (88), it was demonstrated that Erastin increased the susceptibility of non-acute promyelocytic leukemia (APL) AML cells to chemotherapeutic drugs cytarabine and doxorubicin in an RAS-independent manner. Erastin-induced ferroptosis activates the JNK and p38 signaling pathways, but not the ERK/MAPK pathway. Low doses of Erastin, in part due to ferroptosis, increased the susceptibility of non-APL AML cells to cytarabine and doxorubicin.

HMGB1 is a transcription factor that is crucial to the etiology and chemotherapeutic resistance of leukemia (89). Wen *et al* (81) demonstrated that HMGB1 is directly involved in Erastin-induced ferroptosis and is a significant regulator of the process. It was reported that the RAS-JNK/p38 pathway is utilized by HMGB1 to regulate Erastin-mediated ferroptosis and the *in vivo* examination of HMGB1 expression levels did not have a significant impact on experimental animals.

DHA, a natural antimalarial compound found in the Chinese herb *Artemisia annua*, has been reported to significantly inhibit the activity of AML cells (82,90). DHA activates AMPK phosphorylation to downregulate the activity of the mTOR/p70S6k signaling pathway, induces autophagy in AML cells, speeds up ferritin degradation, increases the size of the unstable iron pool, increases cell ROS accumulation and ultimately causes ferroptosis in AML cells.

TYP is a major flavonoid compound extracted from typha pollen. Zhu *et al* (83) reported that TYP serves a significant role in inhibiting the proliferation of AML cells by promoting the activation of the AMPK signal, inducing significant autophagy of AML cells and ultimately causing ferritin degradation, ROS accumulation and ferroptosis. They reportedly assessed the toxicity of TYP by observing weight changes and pathological changes in major organs such as the liver, spleen, kidney and lungs in mice during treatment. The results showed that no weight loss or pathological changes in major organs were observed in mice treated with TYP, indicating a low level of toxicity and good safety.

An all-trans retinoic acid (ATRA) derivative known as ATPR, which was designed and synthesized by Du *et al* (84), exhibits more potent anticancer properties compared with ATRA. ATPR-mediated induction acts through the regulation of iron homeostasis and ROS levels by inhibiting the expression of Nrf2. Nrf2 is an important antioxidant reaction factor and its inhibition results in an increase in the sensitivity of cells to oxidative stress, which promotes autophagy (91). In addition, ATPR also regulates iron homeostasis by increasing ROS levels, a mechanism that may involve the degradation of ferritin and iron metabolism-related proteins, thereby releasing bound iron and increasing LIP and ROS levels, further inducing ferroptosis (92).

Imetelstat is a small oligonucleotide inhibitor of telomerase, a ribonucleoprotein complex that protects and prolongs the telomeres at the end of chromosomes, a process that is involved in cellular senescence and cellular aging (85). Imetelstat acts as a potent inducer of ferroptosis, by promoting excessive lipid peroxidation and oxidative stress in AML, via regulation of PUFA metabolism, which is itself mediated by ACSL4 and FADS2. The preclinical efficacy of imetelstat was evaluated using an AML patient-derived xenograft model. The combination of imetelstat and standard induction chemotherapy, consisting of cytarabine and anthracycline, induced oxidative stress, causing AML cells to become sensitive to imetelstat-induced lipid peroxidation and ferroptosis, which increased the efficacy of chemotherapy on AML. This demonstrated that imetelstat could effectively reduce the burden of AML and delay the recurrence following oxidative stress-induced chemotherapy (85).

Honokiol is a bioactive bisphenol phytochemical that can be isolated from the bark, seed balls and leaves of trees belonging to the genus *Magnolia* (86). It has potent antioxidant, anti-inflammatory, anti-angiogenic and anticancer properties. Notably, honokiol triggers ferroptosis in AML cells by increasing the expression levels of HMOX1, and furthermore, zinc protoporphyrin, an HMOX1 inhibitor, prevented the honokiol-induced ferroptosis of several AML cell lines (THP-1, U-937 and SKM-1 cells) (86). However, the potential of honokiol as a broad-spectrum antileukemic therapy remains to be determined.

In recent years, remarkable progress has been made in the study of drugs targeting ferroptosis drugs for AML. These drugs include small molecule inhibitors, natural compounds and drugs prepared using nanotechnology, which have demonstrated potential therapeutic effects in clinical trials (Table II). Nevertheless, the value of these drugs compared with standard AML treatments still requires further evaluation. Future studies should focus on the safety, efficacy and long-term effects of these drugs in clinical use to determine their practical use in the treatment of AML, with the goal of improving the accuracy of prognostic predictions and developing personalized treatment plans. The varying sensitivities of different subgroups of patients with AML to various treatments, as well as individual differences between patients should be taken into consideration in further research. This research will be of notable significance to improve the survival rates and quality of life of patients.

## 5. Ferroptosis and the prognosis of AML

Dysregulated maturation and differentiation of hematopoietic stem cells and malignant cloning are associated with the formation and progression of AML, a heterogeneous hematological disease (93). The clinical effectiveness in patients with AML was significantly increased following the optimization of targeted treatment and hematopoietic stem cell transplantation, specifically reflected in the significant increases in complete remission rates and overall survival. However, the long-term survival of patients is still limited and patient prognosis remains poor. The association between ferroptosis-related genes (FRG) and the prognosis of AML has garnered interest, due to interest in ferroptosis as a possible therapeutic target for the management of cancer. To improve patient risk adaptation therapy, it is necessary to investigate the prognostic significance of FRGs by establishing a clinical prognostic model for predicting survival risk in patients with AML (Table III).

A previous study showed the normalized levels of each FRG and the regression coefficients were used to create the AML risk score, which was based on the sum of numerous clinical variables (94). The normalized level of each FRG and its regression coefficient were used to calculate the AML risk score. Then, based on a median risk score, patients with AML were split into low-risk and high-risk groups. According to the survival analysis, the mortality rate in the low-risk group was significantly reduced and overall survival was significantly increased. Cox regression analysis was used to develop a combined risk score using the clinical characteristics of patients with AML using data from TCGA, such as age and sex (95). The prognostic risk score model was employed as a prognostic factor independent of other clinical parameters to successfully guide prognosis prediction, based on the multivariate Cox regression analysis, but it has not yet been implemented in clinical practice (96). Another study on the prognosis of two FRGs, DNAJ heat shock protein family member B6 (DNAJB6) and HSPB1, showed they were favorably and adversely correlated with the prognosis of patients with AML, respectively, in a prognostic model created using copy number variation (CNV)-driven FRGs (96). A total of eight ferroptosis regulators PGD, ACSF2, CISD1, DPP4, GPX4ADDIN, SQLE, AIFM2 and CHAC1] were used to create a predictive model, and were all associated with poor prognosis in patients with AML (97,98). In another study, ZFPM2, ZNF560, ZSCAN4, HMX2, HRASLS, LGALS1, LHX6, CCL23 and FAM155B were high-risk genes for prognosis in patients with AML in the prognostic model created using 18 regulators of ferroptosis (99), whereas MXRA5, PCDHB12, PRINS, TMEM56, TWIST1, ASTN1, DLL3, EFNB3 and FOXL1 were genes associated with a favorable prognosis. Another prognostic risk model for AML based on 12 FRGS, including 10 high-risk genes (GPX4, CD44, CISD1, SESN2, LPCAT3, AIFM2, AKR1C2, SOCS1, ACSL5 and HSPB1) and two protective genes (ACSL6 and G3BP1) (100), showed that these genes served an important role in regulating ferroptosis and tumor development (101). *In vitro* research demonstrated that HIVEP3 was a factor in ferroptosis. Using a LASSO model, the integration of HIVEP3 with AIFM2 and LPCAT3 increased the precision of HIVEP3 for predicting a worse prognosis in patients with AML (102). In recent years, the function of these genes and their importance in cancer prognosis have become a focus of increased research. These studies contribute to the understanding of the molecular mechanisms of cancer and may guide future treatment strategies.

DNAJB6 acts as a molecular chaperone protein that functions with Hsp70 to ensure the correct folding of proteins (103). The naturally increased expression of DNAJB6 has been found to decrease GPX4 activity, leading to an increase in ferroptosis. In certain types of cancer, such as esophageal squamous cell carcinoma, downregulated DNAJB6 expression levels have been associated with anticancer effects. Conversely, in AML, low DNAJB6 expression levels were associated with an improved prognosis, which suggested that it may act as a protective factor (104). DNAJB6 is a member of the small heat shock protein family, and as such, HSPB1 is involved in regulating cytoskeletal organization and preventing the aggregation of abnormally folded proteins (105). In breast cancer, non-small cell lung cancer, gastric cancer, and prostate cancer, aberrant expression levels of HSPB1 have been associated with aggressive tumor behavior, chemotherapy resistance, and poor prognosis. However, in AML, HSPB1 expression levels are downregulated and are considered a negative prognostic factor, based on hazard ratio analysis. The phosphorylated form of HSPB1 has been shown to inhibit apoptosis and induce autophagy, while reducing cellular iron uptake and lipid ROS production, which may serve protective roles in AML (106).

As an antioxidant enzyme, AIFM2 operates in conjunction with GPX4 and GSH to inhibit phospholipid peroxidation and prevent ferroptosis (95,107). In certain types of cancer, such as cervical cancer and hepatocellular carcinoma, the expression levels of AIFM2 have been associated with a reduction in tumor formation (108). As a crucial rate-limiting enzyme in cholesterol metabolism, SQLE activity is positively correlated with the proliferation and metastasis of various types of cancers and may impact tumor prognosis (109). Its high expression is typically associated with poor prognosis. The enzyme PGD mediates the pentose phosphate pathway and is often upregulated in certain types of tumors, such as glioblastoma and breast cancer. It potentially promotes the proliferation and survival of tumor cells by modifying their energy metabolism (110). ACSF2 is an acyl-CoA synthetase whose role in AML is unknown, but has been found to correlate with prognosis in other diseases. For example, in hepatocellular carcinoma, high expression of ACSF2 is associated with shorter overall survival and relapse-free survival, predicting worsening prognostics. In contrast, in diabetic nephropathy, increased expression of ACSF2 is associated with tubular damage and predicts disease progressibility. The mechanisms and effects of this enzyme in different diseases are still being studied, but its importance in prognosis has attracted widespread attention (96,111). CHAC1 is suggested to regulate ferroptosis by influencing intracellular GSH levels (112). CISD1 participates in intracellular iron accumulation and oxidative damage, and may potentially affect ferroptosis (113). Specifically, CHAC1, as part of the endoplasmic reticulum stress response pathway, induces ferroptosis by regulating glutathione depletion, promoting intracellular iron accumulation and lipid peroxidation. DPP4 has been suggested to regulate ferroptosis by impacting membrane-associated lipid peroxidation processes. GPX, which functions as an antioxidant enzyme, directly inhibits lipid peroxidation and prevents ferroptosis (114).

Twist-related protein 1 (TWIST), as a pivotal factor in cell transformation, plays a crucial role in the process of normal cells becoming cancerous. Studies have reported that its activity can regulate the cell cycle process in AML cells, enhancing their responsiveness to chemotherapy drugs and increasing the sensitivity of AML patients to treatment, ultimately improving prognosis. Research on TWIST1 in AML primarily focuses on laboratory studies conducted in cell and animal models (115). As an unconventional ligand in the Notch signaling pathway, the upregulation of DLL3 may exert a regulatory effect on the growth and division process of AML cells, which in certain cases is associated with improved survival (116). For instance, in SCLC, high expression of DLL3 has been linked to the responsiveness of certain treatments. Specifically, DLL3-targeted therapies, such as antibody-drug conjugates and T-cell engagers, have shown promising efficacy in clinical trials against tumors with high DLL3 expression (117). LGALS1 is commonly associated with the immunomodulatory function of cells. Research has shown that its expression may support AML cells in evading immune system surveillance and may be linked to increased drug resistance, leading to a poor prognosis (118,119).

Studies have confirmed that PHKG2′s role in regulating polyunsaturated fatty acid peroxidation could impact the sensitivity of cells to ferroptosis inducers, such as Erastin, and potentially affect the ferroptosis process (120). HSD17B11, an enzyme that participates in the reduction or oxidation of sex hormones, may be involved in the regulation of ferroptosis in RSL3-resistant cells (121). Six-transmembrane epithelial antigen of prostate 3 (STEAP3), a metal reductase, can convert iron from Fe^3+^ to Fe^2+^ and is involved in the transcription of cell death genes and regulation of ferroptosis, particularly through its role in p53-mediated processes (122). HRAS, an important member of the cell signaling network, may enhance the sensitivity of AML patients to cytarabine (123). Through high-throughput drug screening and single-cell genomic analysis, studies have found that HRAS mutations are associated with the sensitivity to certain drugs, such as cytarabine (124). These studies suggest that HRAS mutations may enhance the response of AML cells to cytarabine by altering cell signaling pathways. However, their impact on ferroptosis inducers may vary between different types of cancers. For example, in pancreatic ductal adenocarcinoma, KRAS mutations are the most common early genetic alterations and are closely associated with ferroptosis sensitivity (125). On the other hand, in NSCLC, patients with KRAS mutations show lower responsiveness to ferroptosis inducers (126). ARNTL inhibits ferroptosis by suppressing EGLN2 transcription and activating the pro-survival transcription factor HIF1A, and upregulation of ARNTL can increase susceptibility to anticancer drugs (127). SLC38A1, a mediator of glutamine uptake and lipid peroxidation metabolism, is important for iron apoptosis and high expression levels of SLC38A1 have been associated with a poor prognosis for patients with AML (128).

LPCAT3, a key player in the ferroptosis mechanism of cells, facilitates the incorporation of PUFAs into phospholipids, which are essential substrates for lipid peroxidation in ferroptosis (129). Inhibition of LPCAT3 reduced lipid peroxidation, which lead to reduced sensitivity of cells to ferroptosis inducers, such as RSL3 and Erastin (130). Consequently, the regulation of LPCAT3 may significantly impact the occurrence of ferroptosis in patients with AML and potentially serve as a novel therapeutic target for AML treatment. These findings provide promising research directions for the treatment of AML.

FRGs are a major focus of current research on the prognosis of patients with AML as an independent prognostic factor. Further research is required to determine whether the evaluation of FRGs paired with other dysregulated molecular mechanisms may increase the accuracy of predictive models.

## 6. Future perspective and conclusion

In contrast with apoptotic, necrotic and autophagic cell death, ferroptosis is an iron-dependent mode of programmed cell death, more recently identified. Since Dixon *et al* (12) initially described ferroptosis in 2012, an increasing body of data has indicated that ferroptosis is directly linked to the incidence, progression and inhibition of several diseases, however research remains limited regarding its role in AML (131).

The present review discusses the primary mechanisms of ferroptosis and its role in the prognosis and targeted therapy of AML. In the study of AML, ferroptosis is considered to be a complex process involving several signaling pathways, including the GSH/GPX4 pathway, iron metabolism, lipid metabolism and AMPK signaling. As AML cells can escape ferroptosis through several pathways, future treatment strategies should target multiple pathways to ensure that ferroptosis can effectively occur. Certain existing compounds, such as APR-246 and ATPR, have shown potential in inducing ferroptosis in AML cells, but the interaction of ferroptosis with other cell death pathways, such as autophagy and chemotherapy resistance, should also be considered. Advances in nanotechnology offer novel opportunities to precisely target AML cells, potentially inducing ferroptosis while reducing adverse reactions. Through the establishment of a genetic prognostic risk model, the search for FRGs that are closely related to AML may help predict the prognosis of AML and may improve the clinical application of ferroptosis treatment. However, the current understanding of ferroptosis mechanisms and drug resistance in AML remains incomplete, and the targeting of ferroptosis, through the use of small molecule inhibitors, natural compounds and drug nano-administration, provides novel research directions and therapeutic possibilities for the treatment of AML.

## Figures and Tables

**Table I.** Factors influencing the association between ferroptosis and AML.

Factor	Effect	Effect on the occurrence and development of AML
TfR1	A membrane protein that when bound to a transferrin-iron complex, promotes the internalization of iron through siderocytosis.	Elevated iron levels in AML cells, coupled with an imbalance in iron metabolism further increase the susceptibility of AML cells to ferroptosis
GPX4	An antioxidant enzyme that uses GSH to reduce lipid peroxides to non-toxic lipols.	GPX4 is upregulated in AML cells, allowing for the effective inhibition of ferroptosis by reducing lipid peroxide levels.
Nrf2	A key regulator of the antioxidant response, Nrf2 is implicated in AML cell survival.	NF-κB activates Nrf2, increasing the antioxidant capacity of AML cells, thus promoting their proliferation and enhancing their resistance to chemotherapy.
PUFA	The peroxidation of PUFAs in the cell membrane results in the production of lipid peroxides.	The abnormally high levels of fatty acid metabolism in AML cells serves an important role in the occurrence and development of AML by influencing lipid metabolism.

AML, acute myeloid leukemia; TfR1, transferrin receptor 1; GPX4, glutathione peroxidase 4; Nrf2, nuclear factor erythroid 2-related factor 2; PUFA, polyunsaturated fatty acid.

**Table II.** Mechanisms by which drugs or target genes induce ferroptosis.

Drug or ferroptosis inducer	Mechanisms
APR-246	Inhibition of GSH synthesis
ALDH3A2	Inhibition of GPX4 activity
circKDM4C	Upregulated p53
GCFN	Inhibition of GPX4 activity
GCMNPs	Inhibition of GPX4 activity
GNPIPP12MA	Inhibition of GPX4 activity
GNRa-CSP12	Inhibition of GSH synthesis
Erastin	Activation of JNK/p38 pathway
HMGB1	Activation of RAS-JNK/p38 pathway
DHA	Activation of AMPK pathway
TYP	Activation of AMPK pathway
ATPR	Inhibition of Nrf2 activity
Imetelstat	Activation of ACSL4 and FADS2 activity
Honokiol	Activation of HMOX1 activity

GPX4, glutathione peroxidase 4; GSH, glutathione; GPX4, glutathione peroxidase 4; Nrf2, nuclear factor erythroid 2-related factor 2; ACSL4, acyl-coA synthetase long chain family member 4; FADS2, fatty acid desaturase 2; AMPK, AMP-activated protein kinase; APR-246, Eprenetapopt; ALDH3A2, aldehyde dehydrogenase 3 family member A2; circKDM4C, circular RNA derived from the KDM4C gene; GCMNPs, poly(lactic acid)-glycolic acid-encapsulated glycyrrhetinic acids; GNPIPP12MA, glutathione-bioimprinted nanoparticles targeting N6-methyladenosine FTO demethylase; GnRA-CSP12, gold nanorods functionalized with chitosan and a 12-mer peptide12; TYP, typhaneoside; ATPR, 4-amino-2-trifluoromethyl-phenyl retinate HMOX1, heme oxygenase 1; HMGB1, high mobility group box 1; DHA, dihydroartemisinin.

**Table III.** High-risk and protective genes associated with ferroptosis.

High-risk genes	Protective genes
HSPB1, CHAC1, CISD1, DPP4, GPX4, AIFM2, SQLE, PGD, ACSF2, ZFPM2, ZNF560, ZSCAN4, HMX2, HRASLS, LGALS1, LHX6, CCL23, FAM155B, CD44, FH, SESN2, LPCAT3, ACSL5, SOCS1, AKR1C2 and SOCS1	DNAJB6, MXRA5, PCDHB12, PRINS, TMEM56, TWIST1, ASTN1, DLL3, EFNB3, FOXL1, ACSL6 and G3BP1

HSPB1, heat shock protein family B (small) member 1; CHAC1, ChaC glutathione specific gamma-glutamylcyclotransferase 1; CISD1, CDGSH iron sulfur domain 1; DPP4, dipeptidyl peptidase-4; GPX4, glutathione peroxidase 4; AIFM2, apoptosis inducing factor mitochondria associated 2; SQLE, squalene epoxidase; PGD, phosphogluconate dehydrogenase; ACSF2, acyl-CoA synthetase family member 2; ZFPM2, zinc finger protein, FOG family member 2; ZNF560, zinc finger protein 560; ZSCAN4, zinc finger and SCAN domain containing 4; HMX2, H6 family homeobox 2; HRASLS, HRAS like suppressor; LGALS1, lectin, galactoside-binding, soluble, 1; LHX6, LIM homeobox 6; CCL23, C-C motif chemokine ligand 23; FAM155B, family with sequence similarity 155 member B; FH, fumarate hydratase; SESN2, Sestrin 2; LPCAT3, lysophospholipid acyltransferase; ACSL5, acyl-CoA synthetase long chain family member 5; SOCS1, suppressor of cytokine signaling 1; AKR1C2, aldo-keto reductase family 1 member C2; DNAJB6, DnaJ HPS family (Hsp40) member B6; MXRA5, matrix remodeling associated 5; PCDHB12, protocadherin beta 12; TMEM56, transmembrane protein 56; TWIST1, twist family BHLH transcription factor 1; ASTN1, astrotactin 1; DLL3, delta like canonical notch ligand 3; EFNB3, ephrin B3; FOXL19, forkhead box L19; G3BP1, G3BP stress granule assembly factor 1.

## Data Availability

Not applicable.

## Abbreviations

GSH: glutathione
GPX4: glutathione peroxidase 4
Nrf2: nuclear factor erythroid 2-related factor 2
AMP: adenosine 5′-monophosphate
AMPK: AMP-activated protein kinase
MVA pathway: mevalonate pathway
HMG-CoA: hydroxymethylglutaryl-CoA
Acetyl-CoA: acetyl coenzyme A
IPP: isopentenyl pyrophosphate
DMAPP: dimethylallyl pyrophosphate
LIP: labile iron pool
FPN: ferroportin
FTH1: ferritin heavy chain 1
FT: ferritin light chain
TRSP: selenocysteine-specific transfer RNA
FSP1: ferroptosis suppressor protein 1
HO-1: heme oxygenase 1
SLC25A28: solute carrier family 25 member 28
TfR1: transferrin receptor 1
NCOA4: nuclear receptor coactivator 4
IREB2: iron responsive element binding protein 2
HSPB1: heat shock protein family B (small) member 1
STEAP3: six-transmembrane epithelial antigen of prostate 3
DMT1: divalent metal transporter 1
Keap1: ECH associated protein
SAT1: spermine N1-acetyltransferase 1
ALOX15: arachidonate-15-lipoxygenase
GLS2: glutaminase
AA: arachidonic acid
AdA: adrenoyl derivatives
PE-AA/AdA: phosphatidylethanolamines
PUFAs: polyunsaturated fatty acids
BECN1: beclin 1
SLC7A11: solute carrier family 7 member 11
ROS: reactive oxygen species
APR-246: Eprenetapopt
ALDH3A2: aldehyde dehydrogenase 3 family member A2
circKDM4C: circular RNA derived from the KDM4C gene
GCMNPs: poly(lactic acid)-glycolic acid-encapsulated glycyrrhetinic acids
TYP: typhaneoside
HMOX1: heme oxygenase 1
HMGB1: high mobility group box 1
DHA: dihydroartemisinin
GNPIPP12MA: glutathione-bioimprinted nanoparticles targeting N6-methyladenosine FTO demethylase
GnRA-CSP12: gold nanorods functionalized with chitosan and a 12-mer peptide12
RSL3: RAS-selective lethal
ATRA: all-trans retinoic acid
ATPR: 4-amino-2-trifluoromethyl-phenyl retinate
FRG: ferroptosis-related genes
DNAJB6: DNAJ heat shock protein family member B6
TWIST: twist-related protein 1
LPCAT3: lysophospholipid acyltransferase
